# FLT3 tyrosine kinase inhibitors synergize with BCL-2 inhibition to eliminate FLT3/ITD acute leukemia cells through BIM activation

**DOI:** 10.1038/s41392-021-00578-4

**Published:** 2021-05-24

**Authors:** Ruiqi Zhu, Li Li, Bao Nguyen, Jaesung Seo, Min Wu, Tessa Seale, Mark Levis, Amy Duffield, Yu Hu, Donald Small

**Affiliations:** 1 Department of Oncology, Johns Hopkins University School of Medicine, Baltimore, MD USA; 2 Department of Hematology, Union Hospital, Tongji Medical College, Huazhong University of Science and Technology, Wuhan, China; 3 Department of Pathology, Johns Hopkins University School of Medicine, Baltimore, MD USA; 4 Department of Pediatrics, Johns Hopkins University School of Medicine, Baltimore, MD USA

**Keywords:** Drug development, Haematological cancer

## Abstract

Tyrosine kinase inhibitors (TKIs) targeting FLT3 have shown activity but when used alone have achieved limited success in clinical trials, suggesting the need for combination with other drugs. We investigated the combination of FLT3 TKIs (Gilteritinib or Sorafenib), with Venetoclax, a BCL-2 selective inhibitor (BCL-2i), on FLT3/ITD leukemia cells. The combination of a FLT3 TKI and a BCL-2i synergistically reduced cell proliferation and enhanced apoptosis/cell death in FLT3/ITD cell lines and primary AML samples. Venetoclax also re-sensitized FLT3 TKI-resistant cells to Gilteritinib or Sorafenib treatment, mediated through MAPK pathway inhibition. Gilteritinib treatment alone dissociated BIM from MCL-1 but increased the binding of BIM to BCL-2. Venetoclax treatment enhanced the binding of BIM to MCL-1 but dissociated BIM from BCL-2. Treatment with the drugs together resulted in dissociation of BIM from both BCL-2 and MCL-1, with an increased binding of BIM to the cell death mediator BAX, leading to increased apoptosis. These findings suggest that Venetoclax mitigates the unintended pro-survival effects of FLT3 TKI mainly through the dissociation of BIM and BCL-2 and also decreased BIM expression. This study provides evidence that the addition of BCL-2i enhances the effect of FLT3 TKI therapy in FLT3/ITD AML treatment.

## Introduction

Acute myeloid leukemia (AML) is defined as a hematological malignancy characterized by clonal expansion of myeloid hematopoietic cells with blocked differentiation. FLT3 mutations occur in about 1/3 of AML patients, resulting in constitutive activation of FLT3 and its downstream signaling pathways including PI3K/AKT, STAT5, and MAPK, leading to uncontrolled cell growth and reduced apoptosis.^1,2^ There are two types of FLT3 mutations: internal tandem duplication mutations in the juxta-membrane domain (FLT3/ITD) and point mutations in the tyrosine kinase domain (FLT3/KD). FLT3/ITD mutations are the most frequent, occurring in ~23% of AML patients and are associated with an increased relapse risk and decreased disease-free survival. In an attempt to improve the cure rate for these AML patients, several potent tyrosine kinase inhibitors targeting FLT3 (FLT3 TKIs) have been developed. Preclinical and clinical studies have demonstrated that FLT3 TKI are able to inhibit the constitutive kinase activity of FLT3 mutations both in vitro and in vivo.^3^ However, the efficacy of FLT3 TKI used as monotherapy in clinical trials is limited. Resistance and relapse in the small fraction of patients who achieve a CR usually occurs within weeks to months due to the emergence of FLT3 resistance mutations or activation of alternative pathways rendering the cells independent of FLT3 signaling. Combinatorial strategies will be clinically important to reverse resistance and improve outcome in FLT3/ITD AML.^4–9^

BCL-2 family members are the central regulators of cell apoptosis. They can be divided into three subtypes: pro-survival relatives such as BCL-2, MCL-1, and BCL-XL; multi-domain apoptosis executioner proteins BAX and BAK; BCL-2 Homology 3 (BH3)-only pro-apoptotic proteins such as BIM, BID, BIK, NOXA, and PUMA.^10^ Among the BH3-only proteins, BIM, BID, and PUMA function as apoptosis activators as they can directly activate BAX and BAK to form oligomers that induce mitochondrial outer membrane permeabilization (MOMP).^11,12^ The other BH3-only proteins are considered apoptosis sensitizers since they cannot directly activate BAX or BAK.^13^ Instead, they can bind to pro-survival BCL-2 members so that BAX and BAK or BH3-only activator proteins previously binding to pro-survival BCL-2 members can then be released. With the identification of the structural interactions between pro-apoptotic and pro-survival BCL-2 family members, small molecules mimicking the function of the BH3-only proteins to target the pro-survival members, termed BH3 mimetics, have been recently developed.^14^ The identification of the functions of different BCL-2 family members, complemented by emerging insights into the structural interactions between pro-apoptotic and pro-survival family members, led to the concept of killing cancer cells by targeting the pro-survival members with small molecules that mimic the function of the BH3-only proteins, now termed BH3 mimetics.

BH3 mimetics bind to pro-survival proteins, such as BCL-2 and MCL-1, releasing pro-apoptotic proteins from them and thus functioning as inhibitors of these pro-survival proteins. Venetoclax (ABT-199) is a BH3 mimetic which selectively binds to BCL-2 and acts as a selective BCL-2 inhibitor (BCL-2i).^15^ Recent clinical studies determined that Venetoclax improved survival in adults with chronic lymphocytic leukemia (CLL) and AML (in combination with azacytidine or decitabine).^16^

In this study, we investigated the combined effects of FLT3 TKIs (Gilteritinib or Sorafenib) with Venetoclax, a selective BCL-2 inhibitor, on eliminating FLT3/ITD AML cells. We demonstrated that combined treatment of Gilteritinib and Venetoclax has strong synergistic effects on inhibiting proliferation and enhancing apoptosis of FLT3/ITD cells, including Molm14, MV4;11, and TKI (Lestaurtinib)-resistant Molm14-R cells lines, as well as primary de novo or relapsed patient AML samples with FLT3/ITD mutations. In addition, Venetoclax re-sensitizes TKI-resistant Molm14-R cells to TKI treatment through consistent inhibition of the MAPK pathway. We further determined that BIM, rather than other BH3-only proteins (BID, BIK, PUMA), plays a central role in enhancing the combined treatment effects. Finally, we carried out three models (Molm14-R cells, FLT3/ITD primary patient BM cells, and murine leukemic FLT3/ITD;Nup98-HoxD13 (FLT3/ITD; NHD13) BM cells) to validate the combination effects of Gilteritinib and Venetoclax on killing FLT3/ITD cells in vivo. Our findings provide evidence that co-administration of FLT3-TKIs and Venetoclax can serve as a treatment option for AML patients with FLT3/ITD mutations, especially those resistant to TKI treatment.

## Materials and methods

### Primary patient samples

Primary samples were acquired upon Johns Hopkins institutional review board (IRB) approval with written informed consent from all patients and healthy volunteers in accordance with the Declaration of Helsinki. In all, 10 AML samples (including three de novo FLT3/ITD, three relapsed FLT3/ITD, two de novo FLT3/KD, and two FLT3/WT samples) were utilized. Mononuclear cells were isolated from the frozen leukemia samples after thawing using Ficoll-Pique Plus reagent according to the manufacturer’s instructions.

### Cell culture

Both cell lines (Molm14, MV4;11, U937, HL-60, and THP-1) and primary cells were cultured in RPMI-1640 medium (Gibco) supplemented with 10% Fetal Bovine Serum (Gemini) and 100 μg/ml penicillin/streptomycin (Life Technologies). Molm14-R (Molm14 cells resistant to 60 nM Lestaurtinib) were cultured in the above medium supplemented with 60 nM Lestaurtinib. BaF3 cells with FLT3 point mutations were generated as previous described.^17^ All cells were cultured at 37 °C with 5% CO_2_.

### Cell proliferation assay

Cell proliferation ability was measured by MTT (3-(4,5-dimethylthiazol-2-yl)-2,5-diphenylltetrazolium bromide) assay according to the manufacturer’s instructions (Roche). In brief, cells were seeded into 96-well plates at a density of 2 × 10^4^ for cell lines and 2 × 10^5^ for primary AML samples. After drug treatment, 10 μl MTT solution was added to each well followed by 100 μl MTT solvent (10%SDS, 10 mM HCL) 4 h later. OD value was measured using a microplate reader (Bio-Rad).

### Apoptosis assay

Briefly, 1 × 10^6^ cells were seeded into a six-well plate for drug treatment. Cells were collected 48 h after incubation and stained with Annexin V and 7-AAD for 15 min followed by flow cytometry analysis using FACSCelesta (BD Biosciences). Results were analyzed using Flowjo software (Version 9.9.3, Tree Star).

### Cell cycle analysis

In all, 5 × 10^5^ cells were seeded into a six-well plate. Cells were collected 24 h after incubation with/without drugs. Cells were fixed with ice-cold 100% methanol and stained with 7-AAD at 25 μg/ml. Analysis was conducted using FACSCelesta (BD Biosciences) and the results analyzed using Flowjo software (Version 9.9.3, Tree Star).

### Cell transfection

Cell transfection was conducted using Amaxa Nucleofector transfection system as per the manufacturer’s instructions. In brief, 2 × 10^6^ Molm14 or Molm14-R cells were transfected with 2 μg siRNA (si-BIM) (ON-TARGETplus Human BCL2L11, SMARTPool, Horizon Discovery) or plasmids (pcDNA3 Flag BIML, #24233, Addgene) using Amaxa Nucleofector Kit V (Lonza), program X-001 on Nucleofector II instrument. In all, 48 h after transfection, expression of target genes was assessed using western blot and cells subjected to subsequent analysis.

### Western blot and co-immunoprecipitation

Total protein was extracted from cells using RIPA Buffer (Sigma-aldrich, USA) supplemented with Complete Mini Protease Inhibitor Cocktail (Roche). Protein concentration was measured using BCA Protein Assay Kit (ThermoFisher Scientific, US). Western blotting analysis and co-immunoprecipitation were performed as previous described.^7^ Antibodies used in these studies were: p-FLT3 (#3463), AKT(#4685), p-AKT(#4060), ERK(#4695), p-ERK(#8544), STAT5(#25656), p-STAT5(#9359), LC3B(#3868), BIM (#2819), p-GSK3(#9327), BAX (#5023), BAK(#12105) (all from Cell Signaling Technology); MCL-1(sc12756), BID(sc514622), NBK/BIK(sc365625), PUMA(sc374223), GSK3(sc-7291), FLT3(S18) (all from Santa Cruz Biotechnology); BCL-2 (551051, Bioscience), and NOXA (ab13654, abcam).

### In vivo mouse experiments

All animal procedures were conducted in accordance with the policy of the Johns Hopkins Animal Care and Use Committee. Transplantation of Molm14-R cells, FLT3/ITD primary patient BM cells, and leukemic FLT3/ITD;Nup98-HoxD13 (FLT3/ITD;NHD13) BM cells was performed as described previously with slight modifications.^18,19^ For details on transplantation, drug administration, and experimental analysis, see supplementary Methods.

### Statistical analysis

Student unpaired two-tailed *t*-test was used for comparison between two groups, while one-way ANOVA analysis was used for three or more groups. Results were presented as mean ± standard deviation (SD). Data were analyzed using GraphPad software and a *P* value < 0.05 was considered statistically significant.

## Results

### FLT3-TKI (Gilteritinib or Sorafenib) and Venetoclax combine synergistically to inhibit the proliferation of FLT3/ITD cell lines and primary FLT3/ITD AML samples

**Fig. 1:**
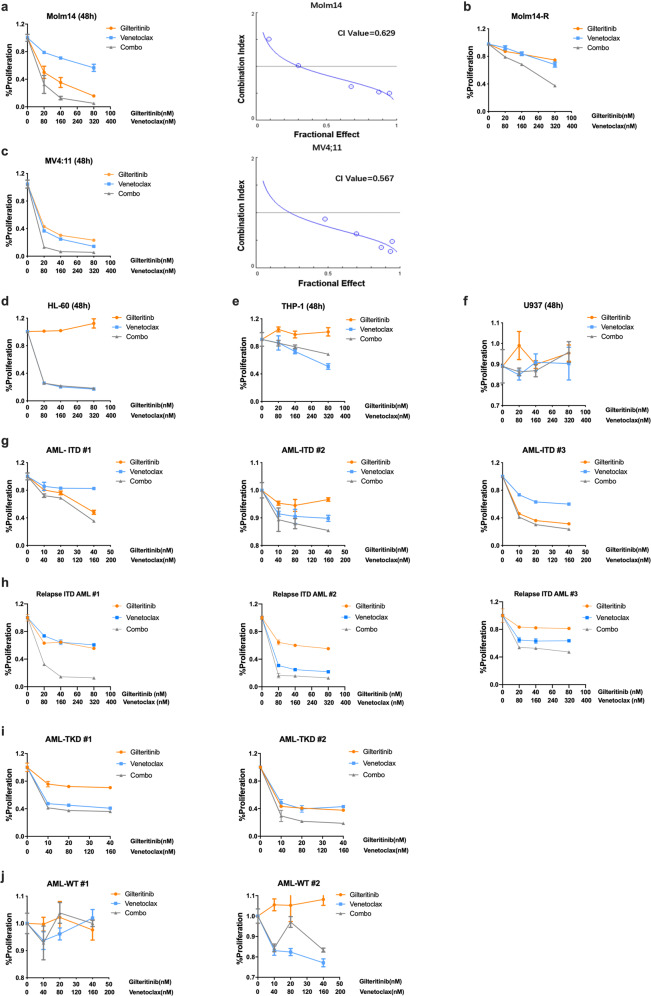
Gilteritinib and Venetoclax synergistically inhibit the proliferation of FLT3/ITD cell lines and primary FLT3/ITD AML samples. 48 h MTT assay of cells treated with the indicated doses of Gilteritinib and Venetoclax: **a** Molm14, **b** Molm14 cells resistant to 60 nM Lestaurtinib (Molm14-R), **c** MV4;11, **d** HL-60, **e** THP-1, **f** U937, **g** Primary de novo AML patient samples with FLT3/ITD mutation (AML-ITD#1, AML-ITD#2, AML-ITD#3), **h** Primary AML samples from relapsed patients with FLT3/ITD mutations (Relapse ITD AML #1, Relapse ITD AML #2, Relapse ITD AML #3), **i** Primary AML patients with FLT3/TKD mutation, or **j** without FLT3 mutations (AML-WT#1, AML-WT#2)

To determine whether combined treatment with a FLT3 TKI and a BCL-2 inhibitor would increase the inhibition of cell proliferation of FLT3/ITD cells, we carried out the MTT assay on Molm14 and MV4;11 cell lines, both of which were derived from AML patients with FLT3/ITD mutations. THP-1, a human FLT3 wild-type (FLT3/WT) cell line, HL-60, a human AML cell line with minimal level of FLT3/WT expression and no response to FLT TKI, as well as U937 cells, a cell line lacking FLT3 expression, were used as controls. As shown in Fig. 1, Molm14 and MV4;11 cells were sensitive to Gilteritinib alone (Fig. 1a, c), with IC_50_s of 20.3 nM and 18.9 nM, respectively, while the other cell lines were insensitive to Gilteritinib (IC_50_s > 100 nM, Fig. 1d–f). Both MV4;11 and HL-60 cells were sensitive to Venetoclax alone, with IC_50_s of 12.5 nM and 7.5 nM, respectively (Fig. 1c, d). The other cell lines, (Molm14, THP-1, U937, and Molm14-R, a substrain of Molm14 cells selected for resistance to the FLT3 TKI Lestaurtinib) were much less or insensitive to Venetoclax treatment (Fig. 1a, b, e, f). A statistically significant synergistic effect was observed for the FLT3/ITD cell lines Molm14, MV4;11, and Molm14-R for the combined treatment with Gilteritinib and Venetoclax for 48 h (*P* < 0.05; Fig. 1a–c). The combinatorial index (CI) values for these cell lines were 0.629 for Molm14, 0.567 for MV4;11, and 0.42 for Molm14-R, respectively. In contrast, no synergy was observed when the FLT3/WT and FLT3 negative control cell lines THP-1, HL-60 and U937 were treated with the combination (Supplementary Table S1; Fig. 1d–f, and Supplementary Fig S1). In addition, to understand whether the synergy also occurs at even earlier time points, we conducted MTT assays in FLT3/ITD cell lines (Molm14, Molm14-R, MV4;11, and MV4;11-R) at 12 and 24 h after treatment. The results showed that 12 or 24 h after treatment with Gilteritinib and/or Venetoclax, the combination group showed synergistic effects on all four FLT3/ITD cell lines (Supplementary Figs. S2 and S3).

We next investigated the effects of the combination of the two drugs on primary AML samples. We observed significant synergy in treating primary AML patient samples containing FLT3/ITD or FLT3/TKD mutations (Fig. 1g, I and Supplementary Fig S4). In contrast, no synergy was observed for combination treatment of primary FLT3/WT AML samples (Fig. 1j). We also observed this synergy when three relapsed AML samples with FLT3/ITD mutations were treated with the combination (Fig. 1h and Supplementary Fig S4). Together, these results suggest that Gilteritinib in combination with Venetoclax is effective in the treatment of primary and relapsed/refractory FLT3/ITD AML.

### FLT3 TKI (Gilteritinib or Sorafenib) and Venetoclax induce apoptosis/cell death of FLT3/ITD cells

**Fig. 2:**
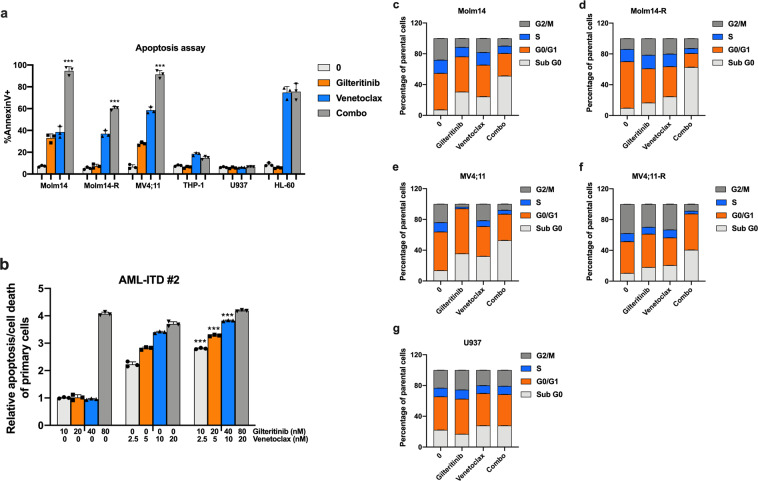
Gilteritinib and Venetoclax induce apoptosis/cell death of FLT3/ITD cells. **a** Bar graph of 48 h apoptosis/cell death assay of Molm14, Molm14-R, MV4;11, THP-1, HL-60, and U937 cells. (Gilteritinib: 80 nM, Venetoclax: 20 nM, Combo: Gilteritinib 80 nM + Venetoclax 20 nM), **b** Bar graph of 48 h apoptosis/cell death assay of primary FLT3/ITD AML patient sample #2 treated with the indicated concentration of Gilteritinib and Venetoclax. **c**–**g** Bar graphs of cell cycle analysis of Molm14, Molm14-R, MV4;11, MV4;11-R, and U937 treated cells

To assess the effect of combination treatment on apoptosis/cell death, we carried out Annexin V-based flow cytometry analysis after drug treatment. After determining effective concentrations, a fixed concentration of Gilteritinib (80 nM) and Venetoclax (20 nM) was used in the apoptosis analysis. Combined treatment resulted in increased apoptosis/cell death of Molm14, MV4;11, and Molm14-R cells compared to either drug alone (*P* < 0.05; Fig. 2a). In contrast, the combination did not increase apoptosis/cell death of HL-60, THP-1, or U937 cells. Similar results were found for the combination of Sorafenib with Venetoclax (Supplementary Fig S5). Increases in apoptosis/cell death were also observed for combination treatment of primary AML samples carrying FLT3/ITD or FLT3/KD mutations (Fig. 2b and Supplementary Fig S6).

We also examined the effect of combination therapy on cell cycling. We examined cells treated with Gilteritinib and/or Venetoclax for 24 h. The results demonstrated that FLT3/ITD cells (Molm14, Molm14-R, MV4;11, and MV4;11-R) had a significantly increased proportion of Sub G0 cells compared to untreated cells or cells treated with either drug alone. No significant changes were found in the proportion of G0/G1, S or G2/M cells (Fig. 2c–g). These were consistent with the data from the apoptosis assay.

### Venetoclax sensitizes TKI-resistant FLT3/ITD cells to FLT3 TKI treatment through inhibiting the MAPK pathway

**Fig. 3:**
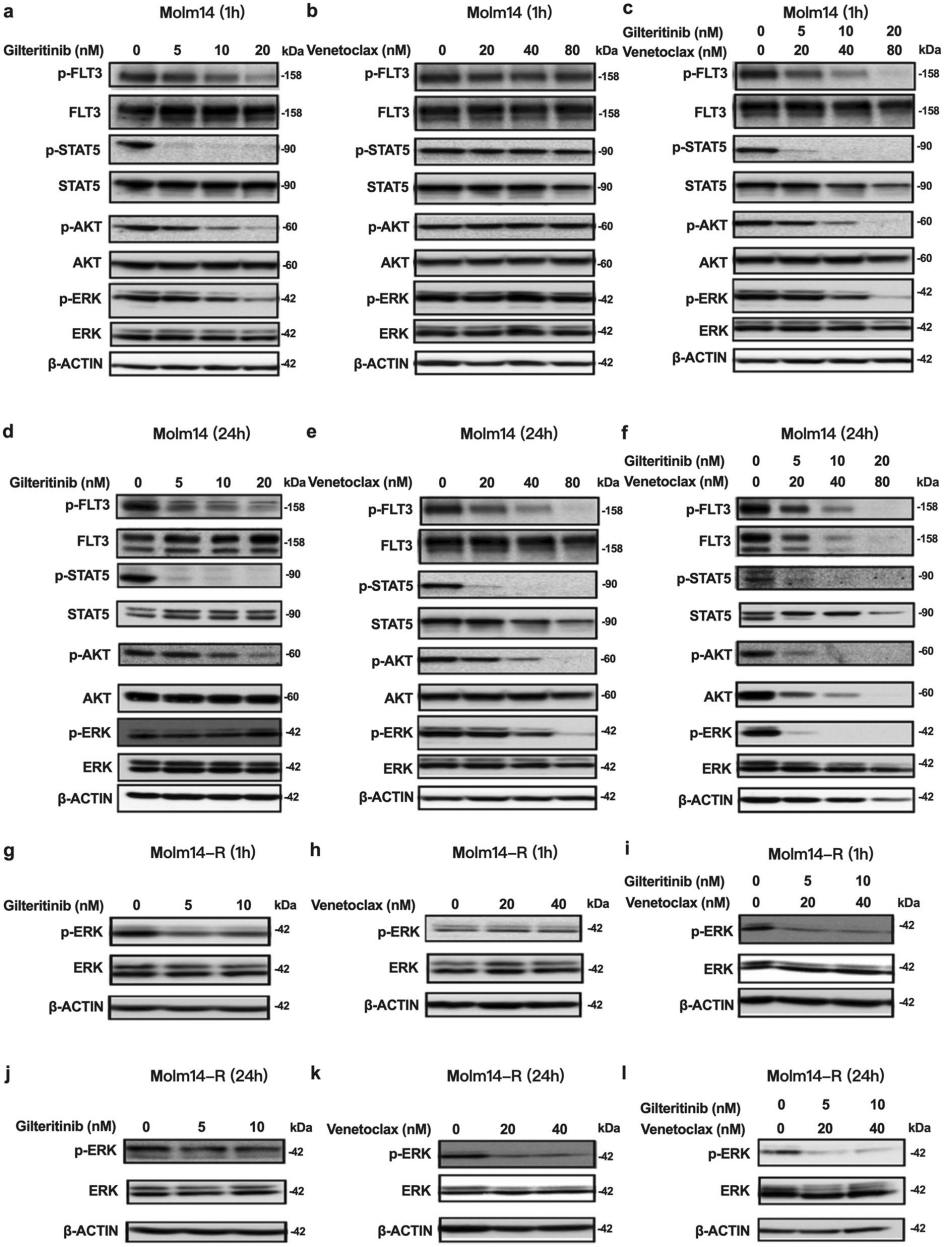
Venetoclax sensitizes TKI-resistant FLT3/ITD cells to TKI treatment through inhibiting the MAPK/ERK pathway. Western blot analysis for FLT3 and downstream signaling pathways in Molm14 cells treated for 1 h with the indicated concentrations of **a** Gilteritinib, **b** Venetoclax, and **c** the combination. In all, 24 h Western blot analysis for FLT3 and downstream signaling pathways in Molm14 cells treated with the indicated concentrations of **d** Gilteritinib, **e** Venetoclax, and **f** the combination. 1 h western blot analysis for FLT3 downstream signaling pathways in Molm14-R cells treated with the indicated concentrations of (**g**) Gilteritinib, (**h**) Venetoclax, and (**i**) the combination. In all, 24 h western blot analysis for FLT3 downstream signaling pathways in Molm14 cells treated with the indicated concentrations of (**j**) Gilteritinib, (**k**) Venetoclax, and (**l**) the combination

To better understand how the combination works together to better kill FLT3/ITD cells, we performed western blot analysis of signal transduction pathways downstream of FLT3. Our previous study had revealed that signaling adaptation in response to FLT3 inhibition results in reactivation of ERK signaling pathways, which contributes to FLT3 TKI resistance.^20^ As shown in Fig. 3a, Gilteritinib effectively inhibited the activity of FLT3 downstream signaling pathways when incubated with Molm14 cells for 1 h. However, by 24 h, despite continued inhibition of FLT3, ERK signaling rebounds as indicated by upregulation of phosphorylated ERK (pERK) (Fig. 3d). Meanwhile, Molm14 cells responded to Venetoclax treatment differently. No changes in FLT3 activity and its downstream signaling were observed after 1 h of Venetoclax treatment. However, by 24 h of treatment the activity of FLT3, STAT5, AKT, and ERK, as assessed by phosphorylation levels, were all reduced, suggesting the inactivation of FLT3 and its downstream signaling with a lag (Figs. 3b, e). Some of the signaling inhibition observed with treatment at 80 nM Venetoclax alone may be due to cell apoptosis/death (Fig. 2a).

Since combined treatment with Gilteritinib and Venetoclax resulted in effective continued suppression of MAPK activity (Fig. 3c, f) in Molm14 sensitive cells, we next examined how the Molm14-R FLT3/ITD cells would respond to the combination treatment. Again, a suppression of p-ERK rebound at 24 h was observed with combined treatment (Fig. 3g–l). The results demonstrate that combined treatment leads to persistent inhibition of the MAPK pathway, suggesting that the addition of Venetoclax prevents the adaptive signaling rebound normally observed after FLT3 TKI treatment and the persistent MAPK signaling seen in resistant cells.

### Gilteritinib and Venetoclax combined treatment releases BIM from the pro-survival proteins BCL-2 and MCL-1 to trigger apoptosis/cell death

**Fig. 4:**
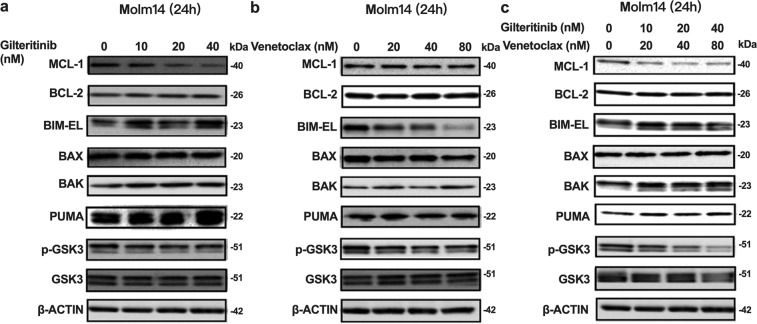
Treatment with Gilteritinib and/or Venetoclax changes expression of some BCL-2 family members. Western blot for BCL-2 family members in Molm14 cells treated for 24 h with the indicated concentrations of **a** Gilteritinib, **b** Venetoclax, and **c** the combination

To determine the mechanism by which the combined treatment with Venetoclax, a BH3 mimetic, results in synergy, we examined how the combination affects expression and interaction of key BCL-2 family members, including MCL-1, BCL-2, BIM, PUMA, BAX, and BAK. Since Gilteritinib and Venetoclax induce increased apoptosis/cell death, it can be difficult to observe expression changes of these proteins, several of which have a short half-life. Based on the Annexin V-based apoptosis results, we treated Molm14 cells with reduced doses of Gilteritinib and Venetoclax which enabled us to follow some of the induced changes. As previously reported, MCL-1 expression decreases when cells are treated with a FLT3 TKI (Fig. 4a).^21^ Combination treatment also reduced MCL-1 expression (Fig. 4a–c). Since MCL-1 upregulation is a key factor for resistance to BCL-2 inhibitors, this decrease in MCL-1 expression in response to FLT3 inhibition may improve the efficacy of BCL-2 inhibition.

BIM is an important apoptosis regulator in hematopoietic cells. We found that Gilteritinib treatment increased BIM expression while Venetoclax treatment had the opposite effect (Fig. 4a, b). Consequently, the combination of the two drugs resulted in neither an increase nor a decrease in BIM expression compared to that of control treated cells (Fig. 4c). PUMA expression was also upregulated by Gilteritinib treatment. No significant differences were observed for expression of BCL-2, BAX, or BAK (Fig. 4a–c).

**Fig. 5:**
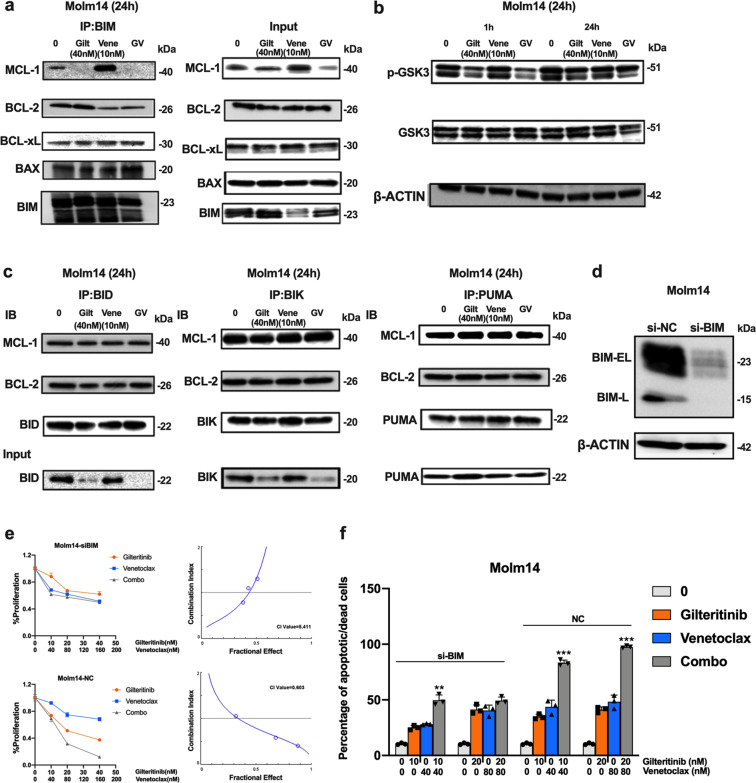
Gilteritinib and Venetoclax combined treatment releases BIM from the pro-survival proteins BCL-2 and MCL-1 to trigger apoptosis/cell death. **a** Co-immunoprecipitation followed by western blot analysis for BIM binding proteins after 24 h of treatment with Gilteritinib and/or Venetoclax. **b** Western blot analysis for p-GSK3 and GSK3 expression in Molm14 cells treated for 1 h and 24 h. **c** Co-immunoprecipitation analysis for BID, BIK, and PUMA binding proteins after 24 h treatment. Input levels of the immunoprecipitated proteins are shown below. **d** Western blot analysis for BIM protein in negative control (si-NC) and si-BIM group. **e** 48 h MTT and **f** 48 h apoptosis analysis for Molm14-si-NC and Molm14-si-BIM cells treated with Gilteritinib and Venetoclax at the indicated concentrations

Accumulating data suggest that during apoptosis, released BIM, as an apoptosis activator, triggers the activation of apoptosis executors BAX and BAK in a “hit-and-run” manner, In this process, rather than forming stable pro-apoptotic complexes, BH3-only activator proteins (BIM, BID, and PUMA) transiently bind with low affinity to the apoptosis executioner proteins BAX/BAK, thus triggering their conformational changes and subsequent oligomerization on the mitochondrial outer membrane and initiating apoptosis.^22^ We thus further investigated the binding of BIM to pro-survival BCL-2 members using co-immunoprecipitation analysis. As shown in Fig. 5a, Gilteritinib decreased the binding of BIM to MCL-1 but increased the binding of BIM to BCL-2. Venetoclax decreased the binding of BIM to BCL-2 but increased the binding of BIM to MCL-1. Combination treatment released BIM from both MCL-1 and BCL-2, leading to increased free BIM available to induce apoptosis. Indeed, combined treatment increased the binding of BIM to pro-apoptotic BAX but did not increase the binding of BIM to pro-survival BCL-XL (Fig. 5a). These results suggest that combination treatment at least partially increases apoptosis through BIM.

GSK3 is a key factor regulating MCL-1 expression whose activation promotes MCL-1 degradation and plays an important role in BCL-2 inhibitor-mediated cell apoptosis.^23,24^ We thus investigated GSK3 activity upon drug treatment in Molm14 cells. p-GSK3 (de-activation of GSK3) decreased after incubation with Gilteritinib for 1 h but rebounded by 24 h of treatment (Fig. 5b). interestingly, the rebound of p-GSK3 was not observed when the Molm14 cells were treated with other FLT3 TKIs (Supplementary Fig S7). Venetoclax alone did not impact the level of GSK3 phosphorylation but combined treatment downregulated p-GSK3β. Activation of GSK3 by the combined treatment implies the possible involvement of GSK3 in inducing apoptosis/cell death. We thus treated cells with the GSK3 inhibitor CHIR-99021 (20 nM) together with Gilteritinib and Venetoclax (CHIRCombo). However, no significant difference was seen in cell proliferation and apoptosis/cell death between the CHIRCombo and Combo only treated (Giltertinib + Venetoclax) group, indicating GSK3 does not mediate Combo-induced cell growth inhibition and apoptosis/cell death in Molm14 cells (Supplementary Fig S8).

Not every BH3-only protein can bind to each of the pro-survival proteins. The binding affinities determine which BH3-only protein(s) participate in drug-induced apoptosis and how the delicate BCL-2 family-member network members interact upon treatment.^25^ BIM has been reported to have great affinity for MCL-1 and BCL-2, with EC_50_s <10 nM. BH3-only proteins, such as PUMA, BID, and BIK, also have high affinity for MCL-1 and BCL-2.^25^ We next investigated whether the binding of BCL-2 and/or MCL-1 to other BH3-only proteins are affected by combined treatment and thus would contribute to the observed synergism. However, co-immunoprecipitation assays did not show changes in the binding between these BH3-only proteins and MCL-1 or BCL-2 upon Gilteritinib and Venetoclax combined treatment (Fig. 5c). These results indicate that BIM, and not PUMA, BID, or BIK, is involved in the apoptosis process induced by the combination treatment.

To further validate that the synergy was mediated through BIM, we knocked down BIM by transfecting siRNA targeting BIM (si-BIM) into Molm14 cells. Western blot confirmed that expression of BIM in the si-BIM-transfected cells was significantly reduced compared with control si-NC-transfected cells (Fig. 5d). The proliferation assay demonstrated that the Gilteritinib/Venetoclax combination no longer showed synergy against the si-BIM-transfected cells (with CI values of 0.603 and 5.411, respectively; Fig. 5e). The higher levels of the Gilteritinib/Venetoclax combination also failed to induce a significant increase in apoptosis/cell death in the si-BIM-transfected cells while the lower dose levels did result in an increase (Fig. 5f). It is possible that the small amount of BIM which escaped the repression by si-BIM transfection, can still bind with BCL-2 and MCL-1. At lower concentrations of the two drugs, this small amount of BIM can be released to induce apoptosis/cell death. However, at higher concentrations of the two drugs, that small amount of BIM was exhausted, and no more BIM could be released to induce apoptosis/cell death, explaining the lack of increase at the higher concentrations. These results further confirm that the synergistic effect of Gilteritinib and Venetoclax on FLT3/ITD cells is mediated through BIM.

### The effects of FLT3 TKI (Gilteritinib or Sorafenib) and Venetoclax treatment on BaF3 cells with FLT3 AL point mutation is related to the mutation types

**Fig. 6:**
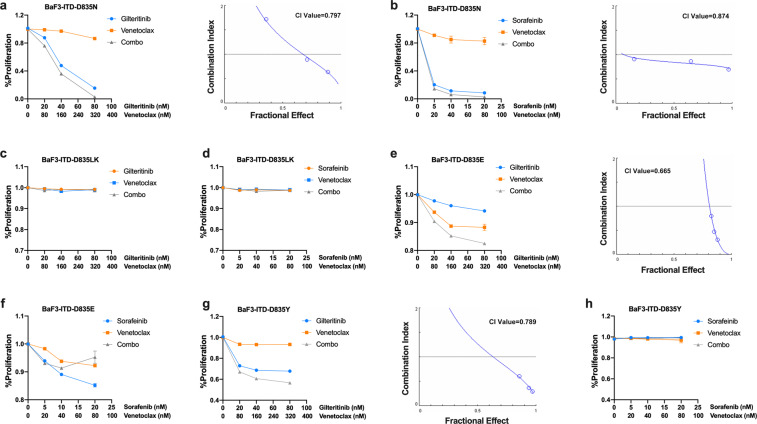
The effects of FLT3 TKI (Gilteritinib or Sorafenib) and/or Venetoclax treatment in BaF3 cells with FLT3 AL point mutations. In all, 48 h MTT assay of **a** BaF3 D835N, **c** BaF3 D835L+K, **e** BaF3 D835E, and **g** BaF3 D835Y cells treated with indicated doses of Gilteritinib and/or Venetoclax. In all, 48 h MTT assay of **b** BaF3 D835N, **d** BaF3 D835L+K, **f** BaF3 D835E, and **h** BaF3 D835Y treated with indicated doses of sorafenib and/or Venetoclax

Although FLT3 TKIs target FLT3-ITD mutation effectively, their effectiveness against the activation loop (AL) mutations of the FLT3 kinase domain varies according to the mutation. We further investigated the synergistic effect of the combination on BaF3 cells with FLT3 point mutations (D835N, D835E, D835Y, and D835L+K) through the proliferation assay. As shown in Fig. 6, synergistic effects of the Gilteritinib/Venetoclax combination were observed for BaF3 D835N, D835Y, and D835E mutations, with CI values (ED75) of 0.797, 0.789, and 0.665, respectively. For the Sorafenib/Venetoclax combination, synergy only occurred for the D835N group (CI value, 0.874). These results suggest that different types of FLT3 AL mutations respond differently to the combination.

Considering that Ba/F3 is a B progenitor cell line and the FLT3 mutations mostly occur in myeloid cells, we further investigated the effects on 32D cells, a myeloid progenitor cell line, carrying FLT3/ITD or FLT3/D835Y mutations (32D-ITD and 32D-D835Y).^26^ The Gilteritinib/Venetoclax combination showed a synergistic effect on the proliferation of both of these cell lines, very similar to the results of the BaF3-ITD and BaF3-D835Y cells (Supplementary Fig. S9).

### Combination of Gilteritinib with Venetoclax effectively eliminates FLT3/ITD AML cells in vivo

**Fig. 7:**
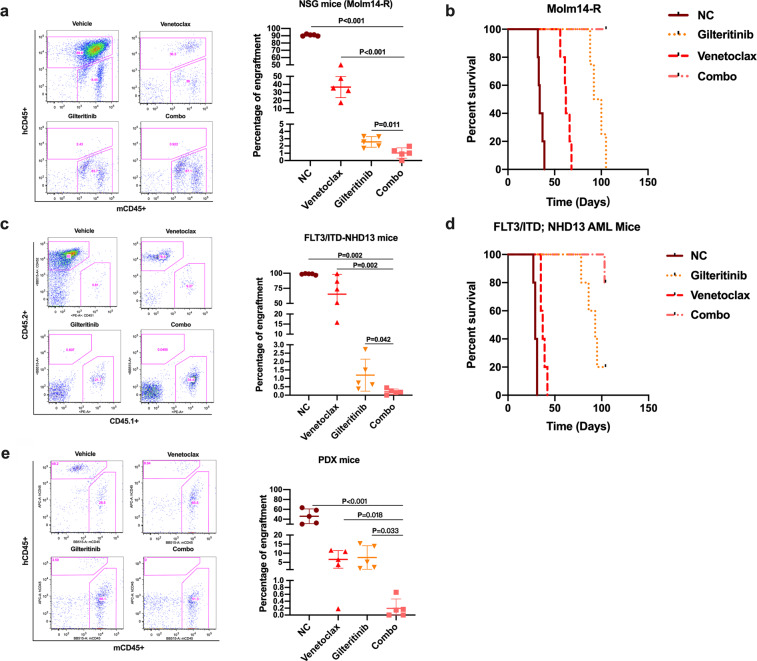
Gilteritinib and Venetoclax combine to more effectively eliminate FLT3/ITD cells in vivo. Mice were treated with vehicle, Gilteritinib (15 mg/kg/day), Venetoclax (80 mg/kg/day), or the combination via oral gavage for various length of time. **a**, **b** Engraftment in BM and Kaplan–Meier survival curve for Molm14-R transplanted mice; **c**, **d** Engraftment in PB and Kaplan–Meier survival curve for FLT3/ITD;NHD13 transplanted mice; **e** Engraftment of human CD45 + cells in the BM of PDX mice

The in vitro data suggests that Gilteritinib and Venetoclax in combination more effectively kill FLT3/ITD cells. To investigate the effects of Gilteritinib and Venetoclax on FLT3 TKI-resistant AML in vivo, we generated a TKI-resistant FLT3/ITD AML model by transplanting lestauritinib-resistant Molm14-R cells into NSG mice. We then treated recipients with Gilteritinib (15 mg/kg/day) and/or Venetoclax (80 mg/kg/day) for 3 weeks. In all, 3 days after the last dosing, we detected various levels of reduction in the fraction of leukemic cells (hCD45+) in the bone marrow of each of the drug-treated cohort (Fig. 7a) compared to that of the vehicle-treated mice. Moreover, the combination of Gilteritinib and Venetoclax showed a significant further reduction compared to either drug alone (Fig. 7a; *P* = 0.011 and *P* < 0.001 compared to Gilteritinib and Venetoclax, respectively). Vehicle-treated mice succumbed to death at a median of 34 days post-transplant while Venetoclax-treated mice survived a median of 62 days. Mice treated with Gilteritinib had a significantly extended median survival of 96 days and mice treated with the Gilteritinib/Venetoclax combination had significantly longer survival beyond 110 days (*P* = 0.0018, 0.0018, and 0.0027 compared with the vehicle control, Venetoclax and Gilteritinib groups, respectively, log-rank test, Fig. 7b).

Next, we evaluated the effects of combination treatment in a model of primary mouse AML by transplantation of spontaneously arising FLT3/ITD;NHD13 (CD45.2+) AML cells from a mouse genetically engineered to have these mutations into syngeneic CD45.1 mice. Mice were treated with Gilteritinib and/or Venetoclax for 3 weeks starting on day 10 after transplantation. Engraftment in recipient PB was assessed when the mouse was dying (before the end time point of the treatment) for vehicle control) or 3 days after the last dosing (for the other three groups). Again, we found that the fraction of mouse leukemic CD45.2+ cells in recipient PB was remarkably lower than those treated with either drug alone or vehicle control (Fig. 7c). Vehicle-treated mice succumbed to death at a median of 29 days post-transplant while Venetoclax-treated mice survived a median of 37 days post-transplant. Mice treated with Gilteritinib had a significantly extended median survival of 93 days. In comparison, Gilteritinib/Venetoclax-treated mice showed a significantly longer survival with only one mouse succumbed to death by the end of the survey (110 days after treatment; *P* = 0.0026, 0.0017, and 0.032 compared with the vehicle control, Venetoclax, and Gilteritinib groups, respectively, log-rank test, Fig. 7d).

In addition, we established a patient derived xenograft (PDX) model with transplantation of BM cells from a primary human de novo FLT3/ITD AML sample to test the effects of the combination treatment. Treatment with Gilteritinib and/or Venetoclax started 60 days after transplantation and continued for 4 weeks. Our results showed that 3 days after treatment, the percentage of human CD45 cells engrafted in the BM was remarkably reduced by combination treatment compared to mice treated with either drug alone or vehicle control (Fig. 7e). These results together strongly suggest that Gilteritinib in combination with Venetoclax have increased efficacy in eliminating FLT3/ITD AML cells in vivo.

## Discussion

Inhibition of pro-survival BCL-2 family members by BH3 mimetics has been shown to be effective in treating patients with AML and lead to the FDA approval of Venetoclax.^27,28^ However, in the trial only 19% of the relapsed/refractory AML patients responded favorably to Venetoclax monotherapy. Thus, combined treatment of Venetoclax with other agents will be necessary to more successfully treat AML. Previous studies have demonstrated that combination of BCL-2 inhibitors with MCL-1 inhibitors,^29^ JAK1/2 inhibitors,^30^ and TKIs^31^ or P53 activators^24^ can synergistically eliminate AML cells.

Constitutive activation of FLT3 by mutation leads to elevated MCL-1 expression,^21,32^ a known mechanism for Venetoclax resistance. Inhibition with FLT3 TKIs can reverse Venetoclax resistance in FLT3 mutant cells.^33^ FLT3 TKIs could effectively downregulate MCL-1 expression, thus re-sensitizing AML cells to Venetoclax treatment.^32,34,35^ In the present study, we demonstrated that Gilteritinib, a recent FDA approved highly effective FLT3 TKI, or the combination of Gilteritinib and Venetoclax significantly downregulates MCL-1 expression. The combination of downregulating MCL-1 expression by Gilteritinib along with BCL-2 inhibition by Venetoclax increased cell death of FLT3 mutant cell lines and primary cells compared with treatment with either drug alone.

The mechanism(s) by which FLT3/ITD cells undergo apoptosis/cell death when treated with a FLT3 TKI and BCL-2i has not been previously shown. Several lines of evidence demonstrated that BIM is an essential regulator of leukopoiesis and plays a central role in Sorafenib-induced AML cell death.^36,37^ Expression of PUMA is also upregulated by FLT3 TKIs.^38^ The interaction between PUMA and BCL-2 or MCL-1 is enhanced when FLT3/ITD cells are treated with Sorafenib plus Clofarabine, Fludarabine, and Busulfan. As BID and BIK are also BH3-only proteins that can bind to MCL-1 or BCL-2 with high affinities, we explored whether these proteins were responsible for the Gilteritinib plus Venetoclax-induced FLT3/ITD cell apoptosis/cell death. By conducting co-immunoprecipitation experiments, we observed that it is BIM and not the other BH3-only proteins (PUMA, BID, or BIK), that dissociates from both MCL-1 and BCL-2 under combined treatment and acts as the apoptosis initiator by binding to BAX/BAK. Similar results were recently reported.^39^ In their study, MCL-1 downregulation and the interaction between BIM and BCL-2/MCL-1 were observed after combined treatment with a FLT3 TKI and BCL-2 inhibition. In addition, they suggested that MAPK/ERK pathway activation caused by Venetoclax treatment is an important reason for Venetoclax resistance in FLT3/ITD cells. However, in our study, we observed significantly decreased levels of ERK activation after treatment with Venetoclax alone. Repression of phospho-ERK by Venetoclax reverses the persistent activation of the MAPK/ERK pathway observed in FLT3-TKI-resistant Molm14 cells, thereby re-sensitizing Molm14-R cells to FLT3 TKI treatment. We thought the perhaps the rebound of phospho-ERK signal reported in the recent report was due to the high concentration of Venetoclax (500 nM) used in their study. To test this possibility, we treated Molm14 cells with Venetoclax at concentrations ranging from 0 to 640 nM. However, ERK activity was still inhibited even at the highest concentration of 640 nM (Supplementary Fig S10). Perhaps the difference observed between these two studies can be attributed to differences in the potency of protein lysis buffer used. In our study, we used RIPA buffer, in which the effective component is IGEPAL CA-630. It is 10-fold more potent than Nonidet P-40,^40^ thus can dissolve membranes of various cell components and release proteins more thoroughly for further detection. Nevertheless, we clearly demonstrated that although BID, BIK, and PUMA are all BH3-only proteins with high affinity for both MCL-1 and BCL-2, BIM and not them, plays a crucial role in the apoptosis/cell death induced by combination treatment.

In conclusion, this study demonstrates that combined treatment with a FLT3 TKI and BCL-2i synergistically inhibits proliferation and induces apoptosis/cell death of FLT3/ITD leukemia cell lines and primary FLT3/ITD AML patient samples, both in vitro and in vivo. BIM, and not the other BH3-only proteins (PUMA, BID, or BIK), plays the key role in the apoptotic process induced by combination treatment. This work supports clinical trials of such combinations in FLT3 mutant AML patients.

## Supplementary information

Supplemental material

## Data Availability

Data supports the results reported in the article can be found in the article and the supplementary materials.
