# Supplementary material for: FLT3 tyrosine kinase inhibitors synergize with BCL-2 inhibition to eliminate FLT3/ITD acute leukemia cells through BIM activation
Source: Signal Transduct Target Ther. 2021 May 24;6:186. doi: 10.1038/s41392-021-00578-4 (PMC8141515; doi:10.1038/s41392-021-00578-4)
Supplement: Supplementary file 1 — Supplemental material [file 41392_2021_578_MOESM1_ESM.docx]

Supplementary Materials for

FLT3 Tyrosine Kinase Inhibitors Synergize with BCL-2 Inhibition to Eliminate FLT3/ITD Acute Leukemia Cells through BIM Activation

Ruiqi Zhu, Li Li, Bao Nguyen, Jaesung Seo, Min Wu, Tessa Seale, Mark Levis, Amy Duffield, Yu Hu, Donald Small

Correspondence to: Donald Small: [donsmall@jhmi.edu](mailto:donsmall@jhmi.edu) Yu Hu: dr_huyu@126.com

**This PDF file includes:**

Supplementary Methods

Supplementary Figures. S1 to S10

Supplementary Table. S1

Supplementary Methods

***In vivo* mouse experiments**

All animal procedures were conducted in accordance with the policy of the Johns Hopkins Animal Care and Use Committee. *In vivo* treatment experiments were performed as described previously ^1,2^ with modifications. In brief, for FLT3/ITD cell line transplantation model, 2×10^5^ Molm14-R cells (human-derived, resistant to 60nM Lestaurtinib) were injected into sublethally irradiated (2.5 Gy) *NOD/SCID-IL-2Rγ^−/−^* (NSG, in-house breeding) mice via lateral tail vein injection. For patient-derived xenograft (PDX) model, sublethally irradiated (2.5Gy) NSG mice were transplanted with 5×10^5^ BM cells isolated from a *de novo* FLT3/ITD AML patient via lateral tail vein injection. For mouse primary FLT3/ITD AML transplantation model, FLT3/ITD;Nup98-HoxD13 (FLT3/ITD;NHD13) mice were generated as previously reported^3^. 5×10^5^ BM cells (CD45.2+) isolated from a FLT3/ITD;NHD13 mouse developing full-blown AML were transplanted into lethally irradiated (6.5Gy) syngeneic Ly5.1 (CD45.1+) mice. Treatment started 7, 10 and 60 days after transplantation for Molm14-R, FLT3/ITD;NHD13 and PDX mice, respectively. These time points were determined based on our previous transplantation experiments under the same transplantation conditions (mouse strain of the recipients, irradiation dosage and donor cells). Mice were treated with vehicle, gilteritinib (15mg/kg), venetoclax (80mg/kg) or combination via oral gavage for 3 weeks (Molm14-R and FLT3/ITD;NHD13 models) or 4 weeks (PDX model). 3 days after treatment or when the mice showed signs of sickness as hunched posture, slow movement and ruffled hair, we collected peripheral blood cells from submendibular vein via cheek bleeding or BM cells via femoral BM aspiration on live recipients. Percentage of donor cells in total mononuclear cells, i.e., human CD45/ (human + mouse CD45) for Molm14-R and primary PDX donor cells and mouse CD45.2 / (mouse CD45.2+CD45.1) for FLT3/ITD;NHD13 donor cells, respectively, was assessed using flow cytometry analysis. Recipients were further monitored for survival. There were five mice in each group.

1 Ma, H. *et al.* TTT-3002 is a novel FLT3 tyrosine kinase inhibitor with activity against FLT3-associated leukemias in vitro and in vivo. *Blood* **123**, 1525-1534, doi:10.1182/blood-2013-08-523035 (2014).

2 Piloto, O. *et al.* Inhibitory anti-FLT3 antibodies are capable of mediating antibody-dependent cell-mediated cytotoxicity and reducing engraftment of acute myelogenous leukemia blasts in nonobese diabetic/severe combined immunodeficient mice. *Cancer research* **65**, 1514-1522, doi:10.1158/0008-5472.Can-04-3081 (2005).

3 Greenblatt, S. *et al.* Knock-in of a FLT3/ITD mutation cooperates with a NUP98-HOXD13 fusion to generate acute myeloid leukemia in a mouse model. *Blood* **119**, 2883-2894, doi:10.1182/blood-2011-10-382283 (2012).


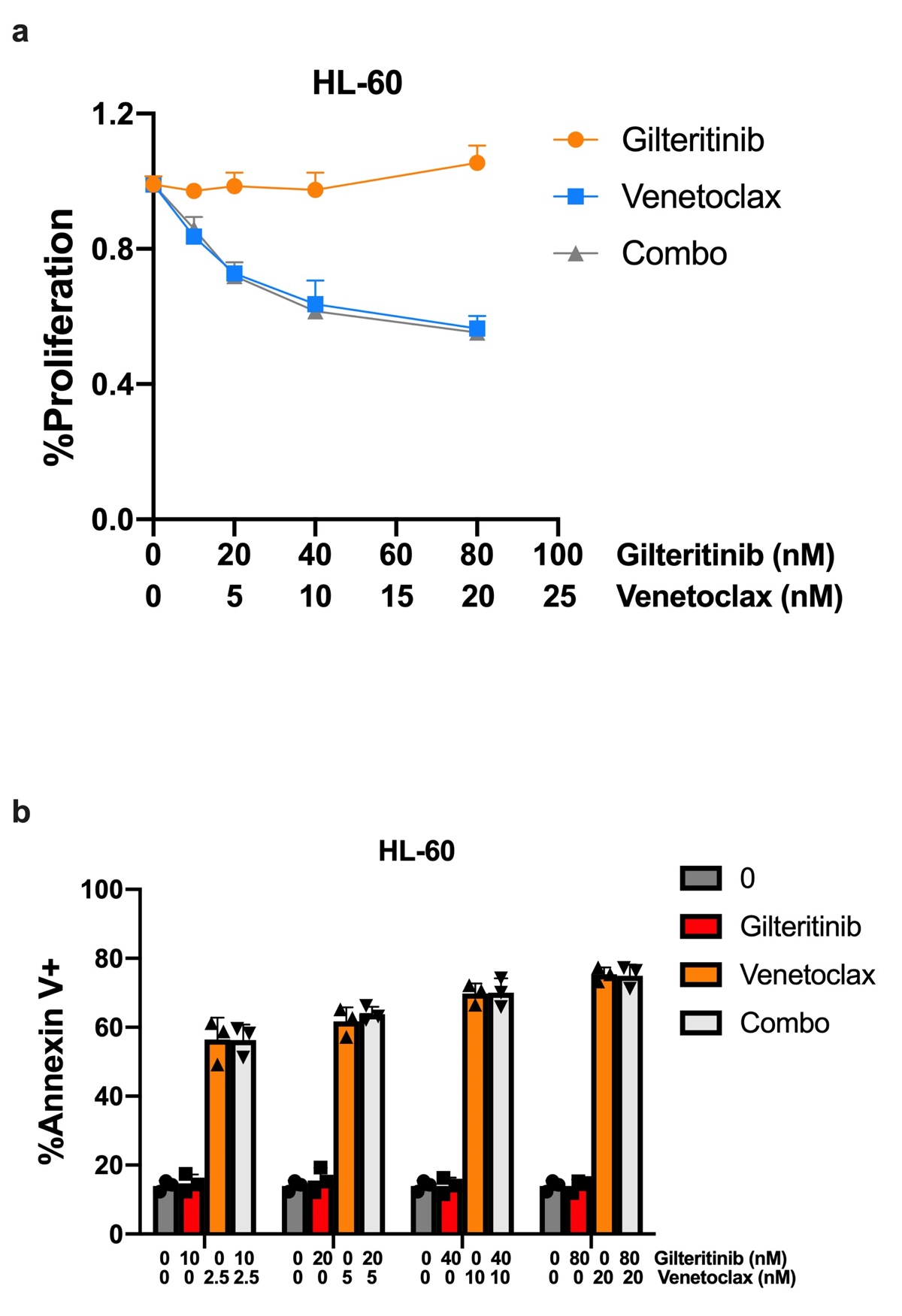


**Fig S1.** No synergism observed for the combination of gilteritinib and venetoclax in HL-60 cells. (a) 48h MTT assay and (b) apoptosis assay of HL-60 cells treated with indicated doses of Gilteritinib and Venetoclax.


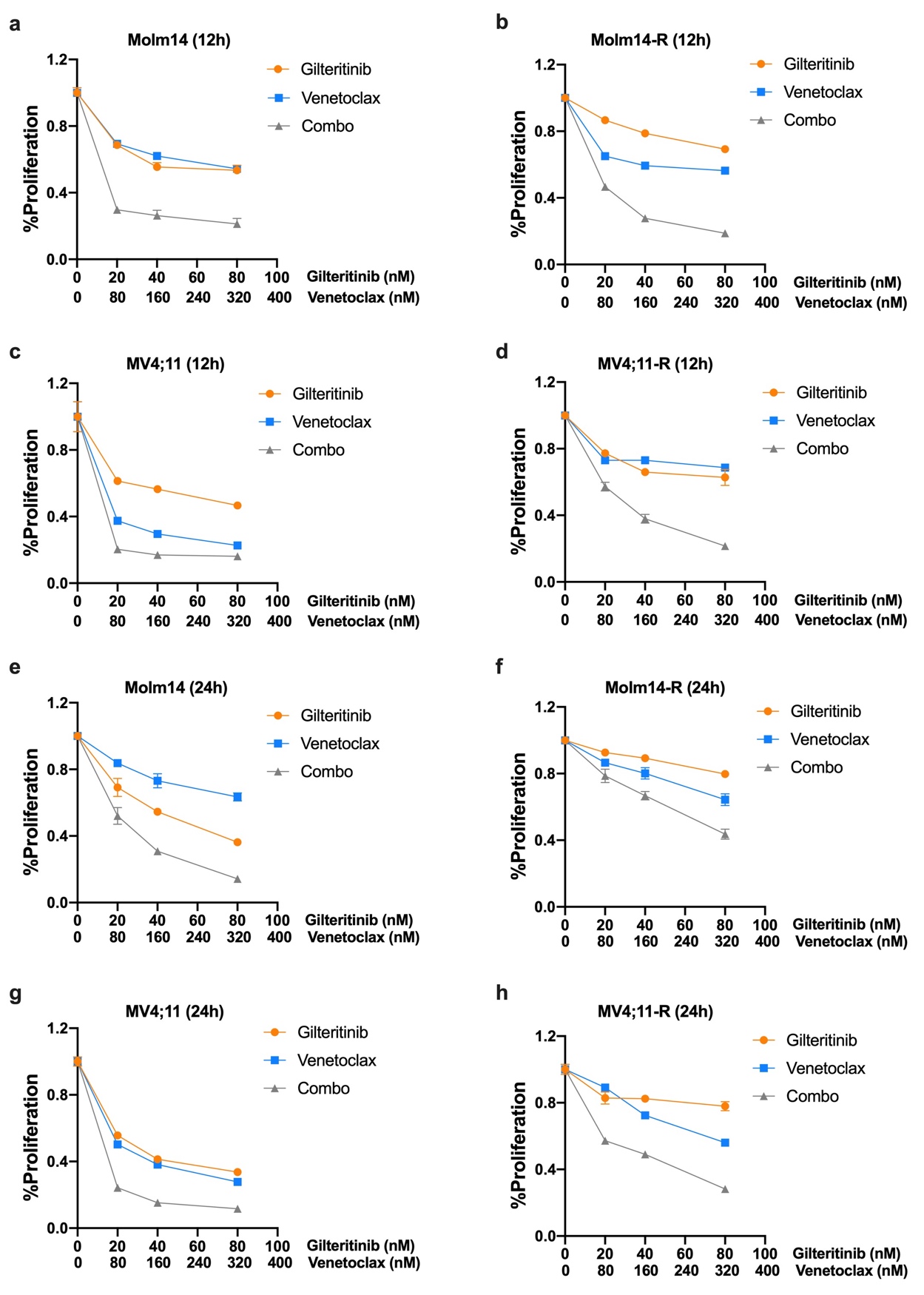


**Fig S2.** Gilteritinib and Venetoclax synergistically inhibit the proliferation of FLT3/ITD cell lines. 12h MTT assay of cells treated with the indicated doses of Gilteritinib and Venetoclax: (a) Molm14 (b) Molm14-R (c) MV4;11 (d) MV4;11-R. 24h MTT assay of cells treated with the indicated doses of Gilteritinib and Venetoclax: (e) Molm14, (f) Molm14-R, (g) MV4;11, (h) MV4;11-R.


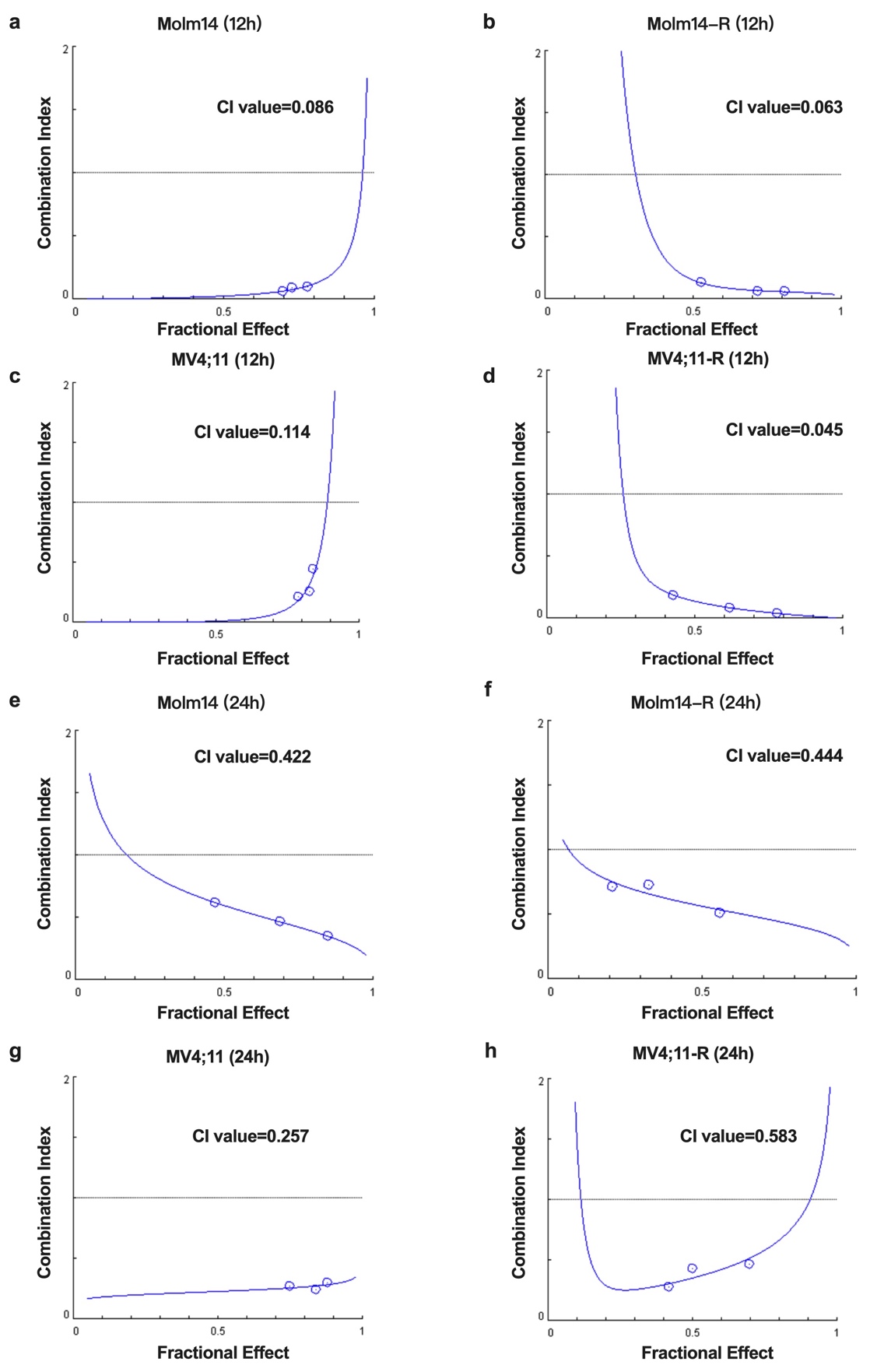


**Fig S3.** Gilteritinib and Venetoclax synergistically inhibit the proliferation of FLT3/ITD cell lines. Combination Index (CI value) images of 12h MTT assay of cells treated with the indicated doses of Gilteritinib and Venetoclax: (a) Molm14 (b) Molm14-R (c) MV4;11 (d) MV4;11-R. CI value images of 24h MTT assay of cells treated with the indicated doses of Gilteritinib and Venetoclax: (e) Molm14, (f) Molm14-R, (g) MV4;11, (h) MV4;11-R.


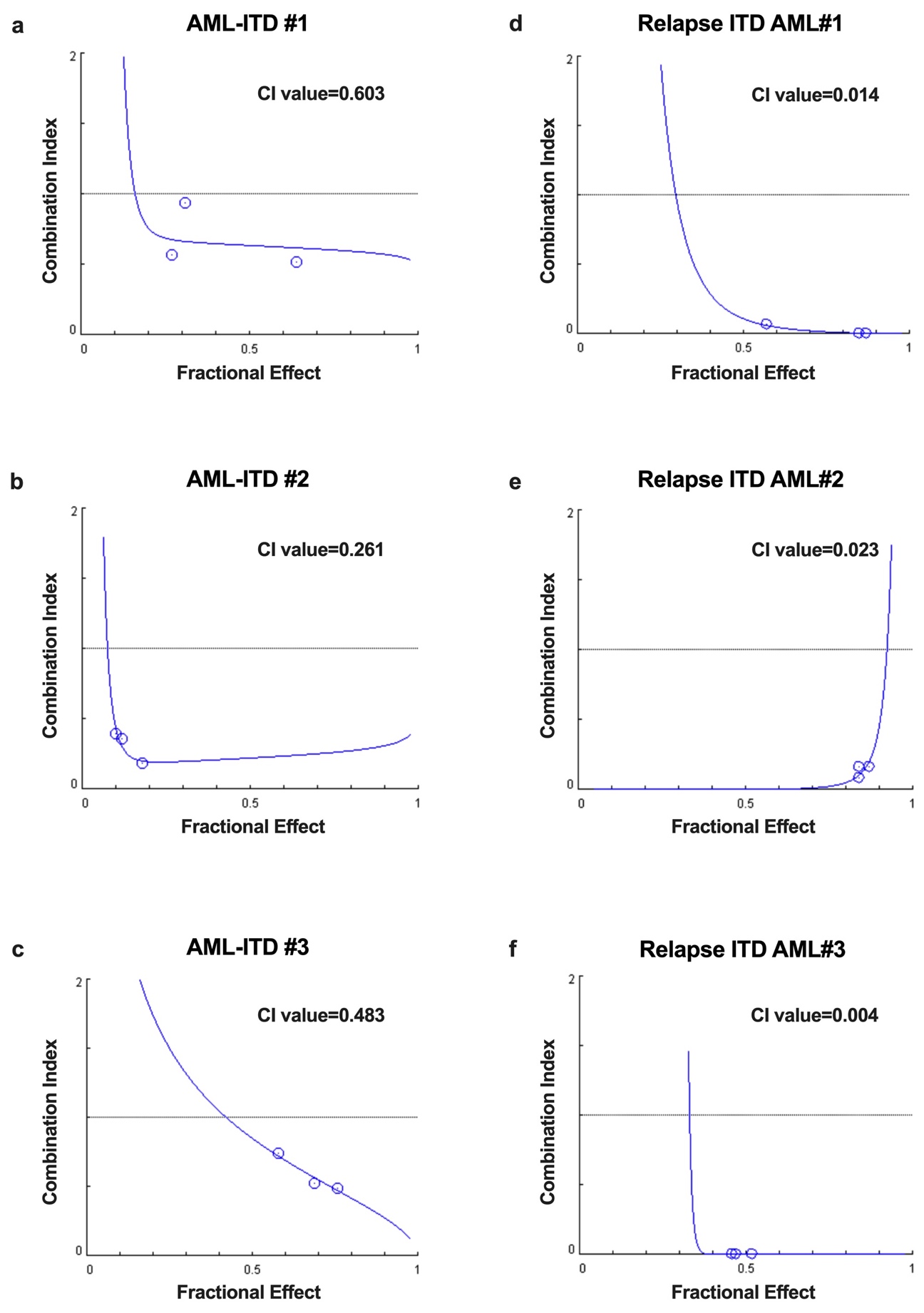


**Fig S4.** Gilteritinib and Venetoclax synergistically inhibit the proliferation of FLT3/ITD AML samples. CI value of (a-c) de novo FLT3/ITD AML samples and (d-f) relapsed FLT3/ITD AML samples.


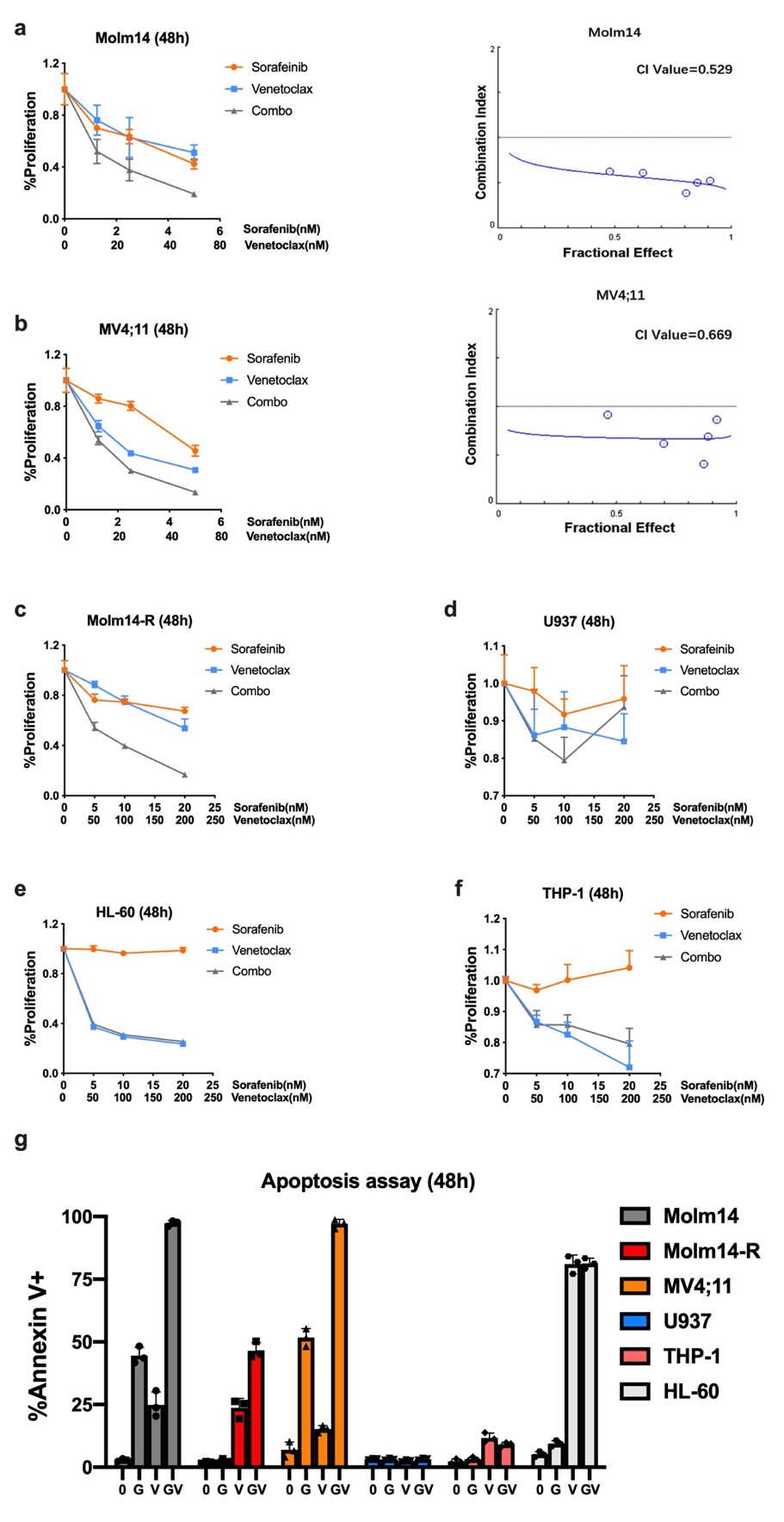


**Fig S5.** Sorafenib and Venetoclax synergistically inhibit the proliferation and increase the apoptosis/cell death of FLT3/ITD cell lines. 48h MTT assay of (a) Molm14 (b) MV4;11 (c) Molm14 cells resistant to 60nM Lestaurtinib (Molm14-R) (d) HL-60 (e) THP-1 (f) U937. (g) Bar graph of 48h apoptosis assay of Molm14, Molm14-R, MV4;11, THP-1, HL-60 and U937 cells.


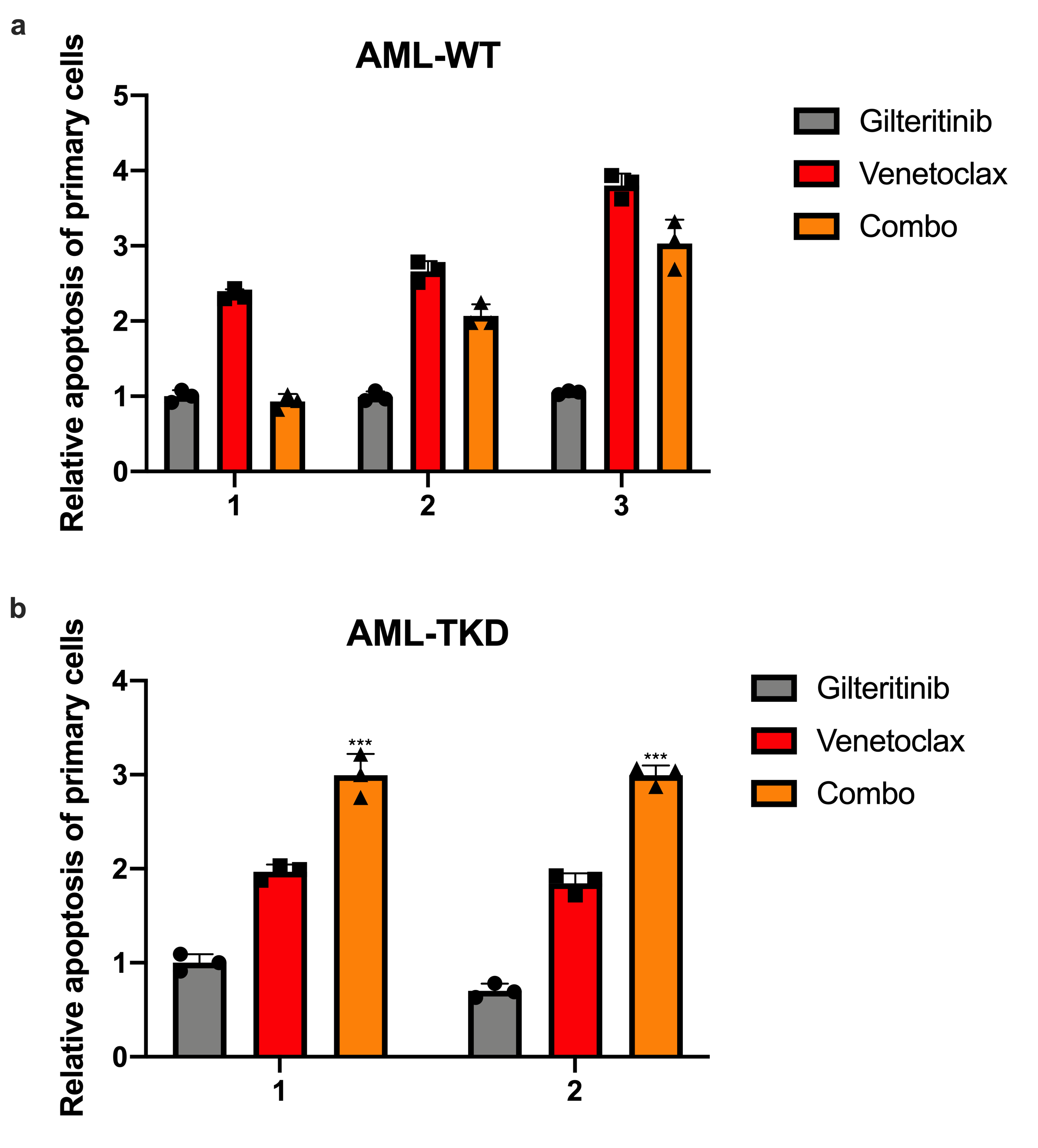


**Fig S6.** AnnexinV/7AAD+ apoptosis assay of (a) 3 FLT3/WT and (b) 2 FLT3/TKD primary patient AML samples treated with Gilteritinib and/or Venetoclax for 48 hours at 80nm and 20nm, respectively.


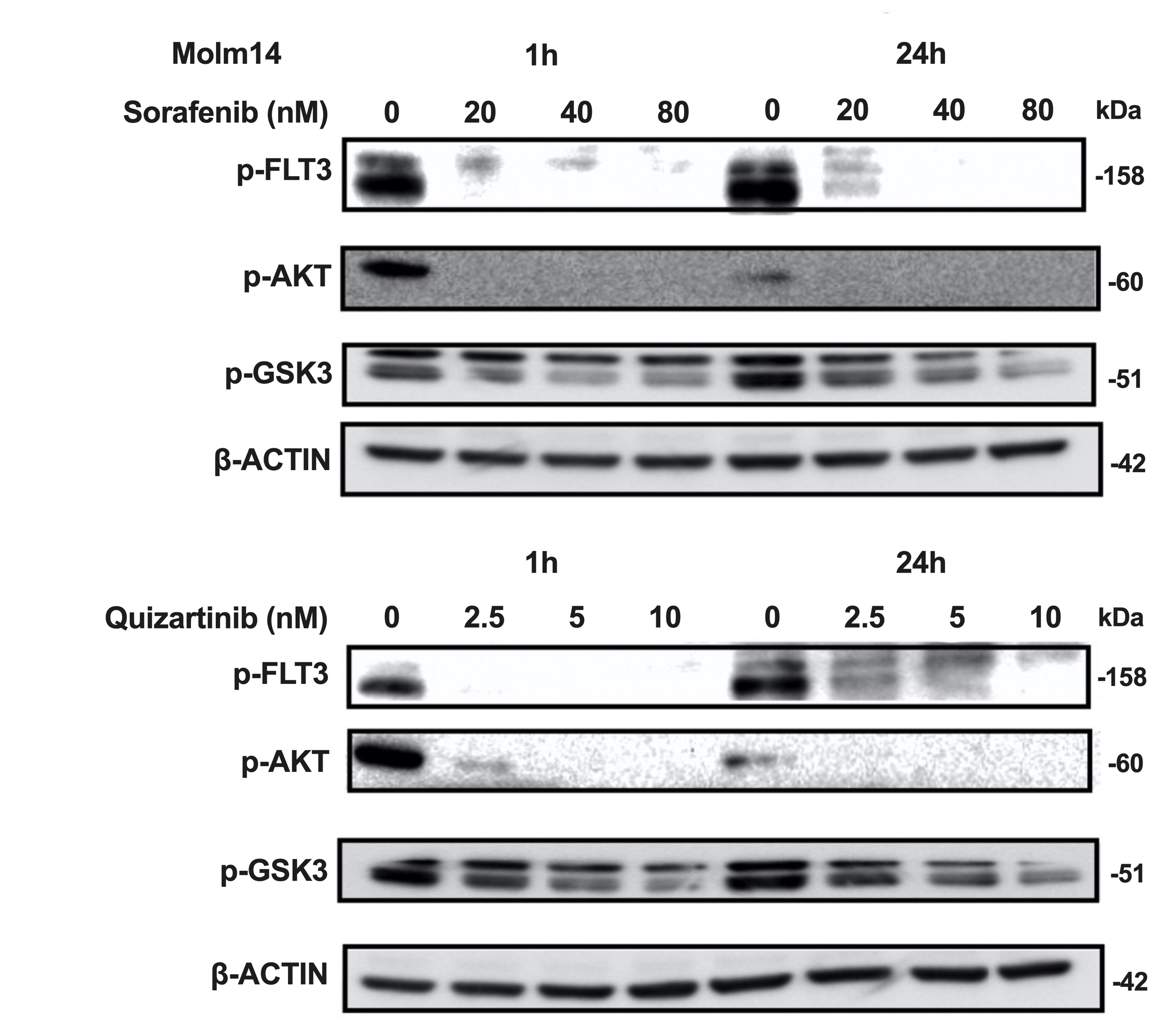


**Fig S7.** Western blot analysis for p-FLT3, p-AKT and p-GSK3 levels in Molm14 cells treated with the indicated concentrations of sorafenib or quizartinib for 1h and 24h. Actin is shown as a control for protein loading.


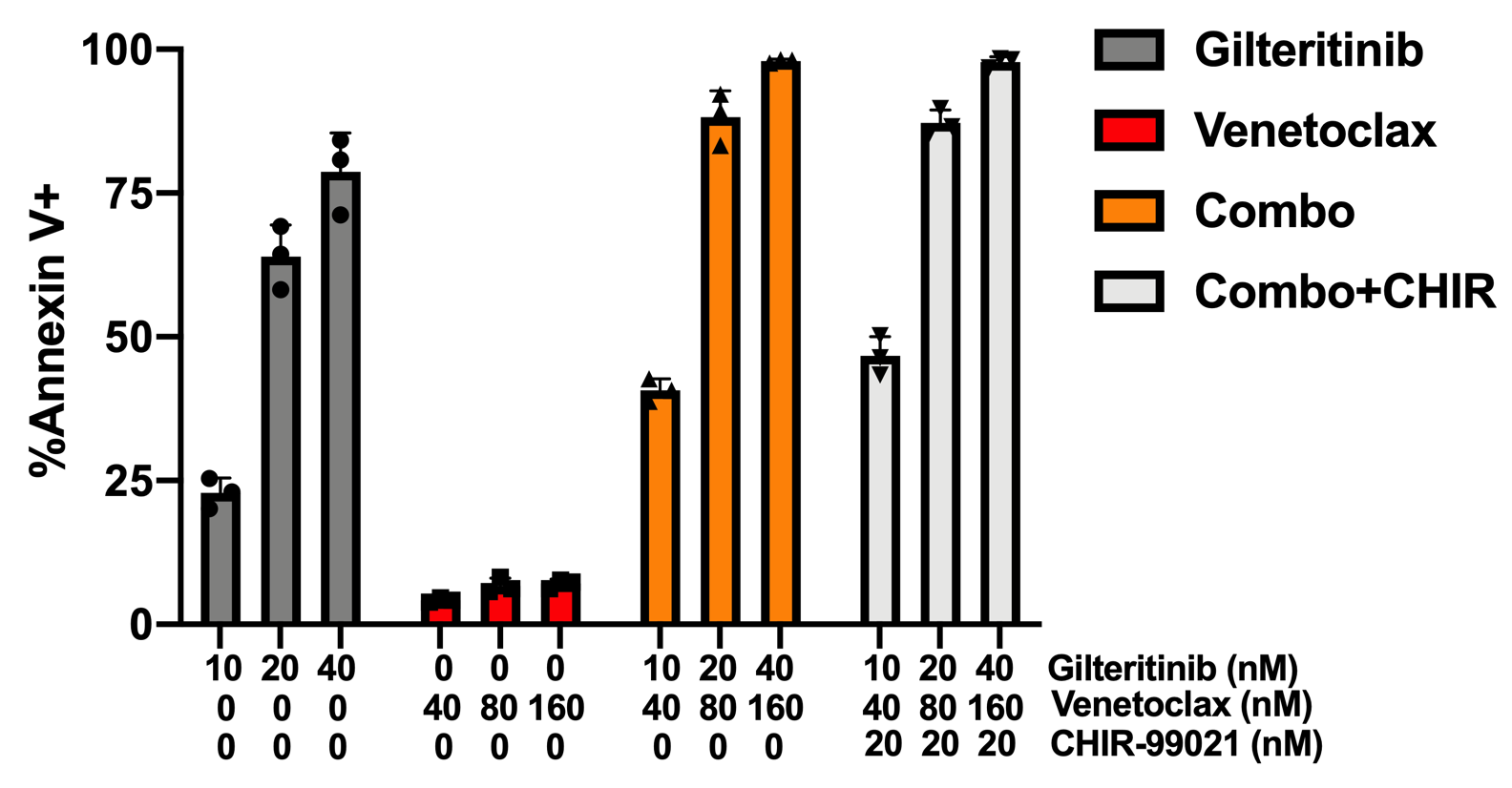


**Fig S8.** AnnexinV+ results for Molm14 cells treated with the indicated concentrations of Gilteritinib, and/or Venetoclax and the GSK3 inhibitor CHIR-99021.

**
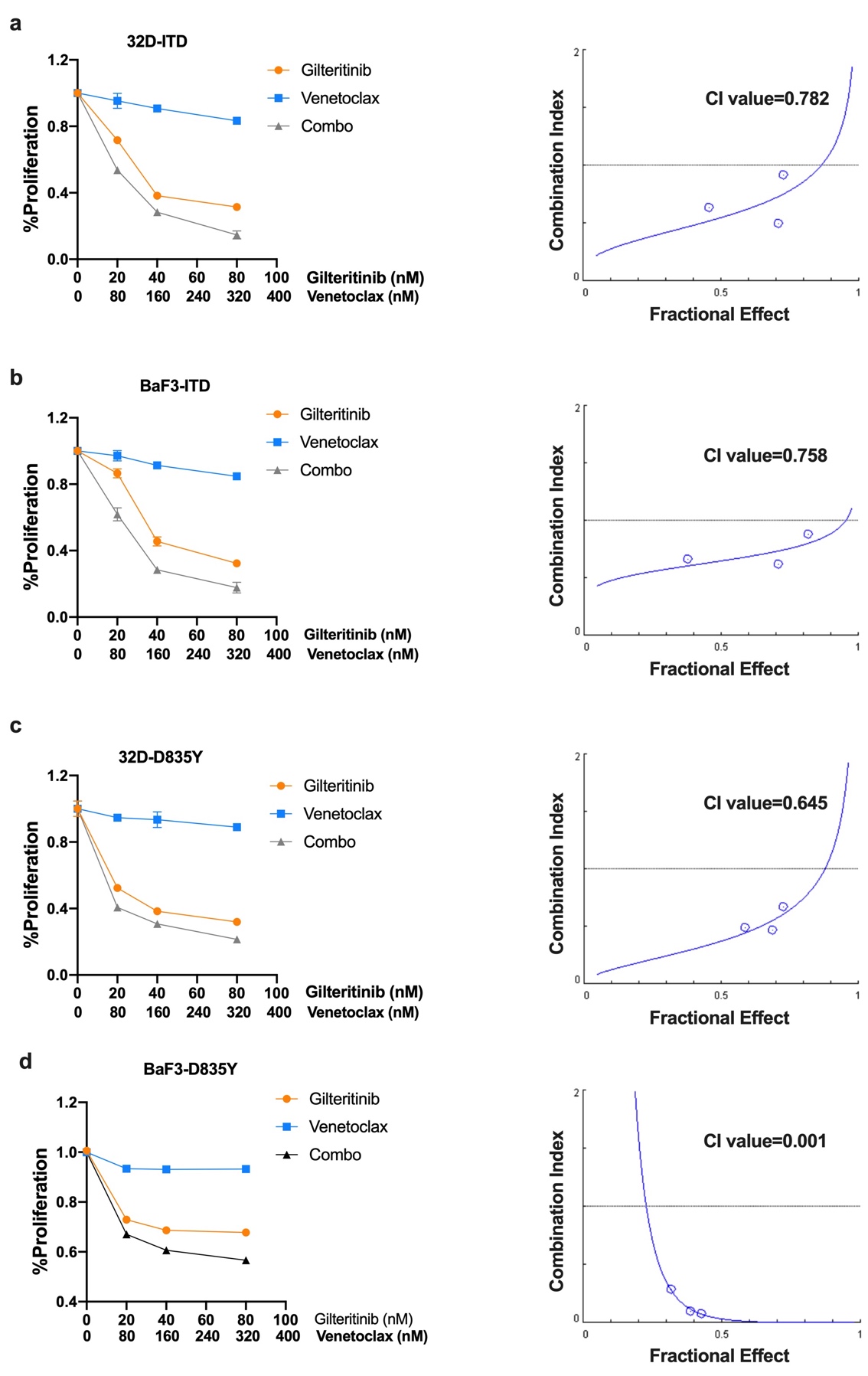
**

**Fig S9.** The effect of combined gilteritinib/venetoclax treatment of 32D-TKD cells are similar to those observed for BaF3-TKD cells. 48h MTT assay and resulting CI values of (a) 32D-ITD, (b) BaF3-ITD, (c) 32D-D835Y and (d) BaF3-D835Y cells treated with indicated doses of Gilteritinib and/or Venetoclax.


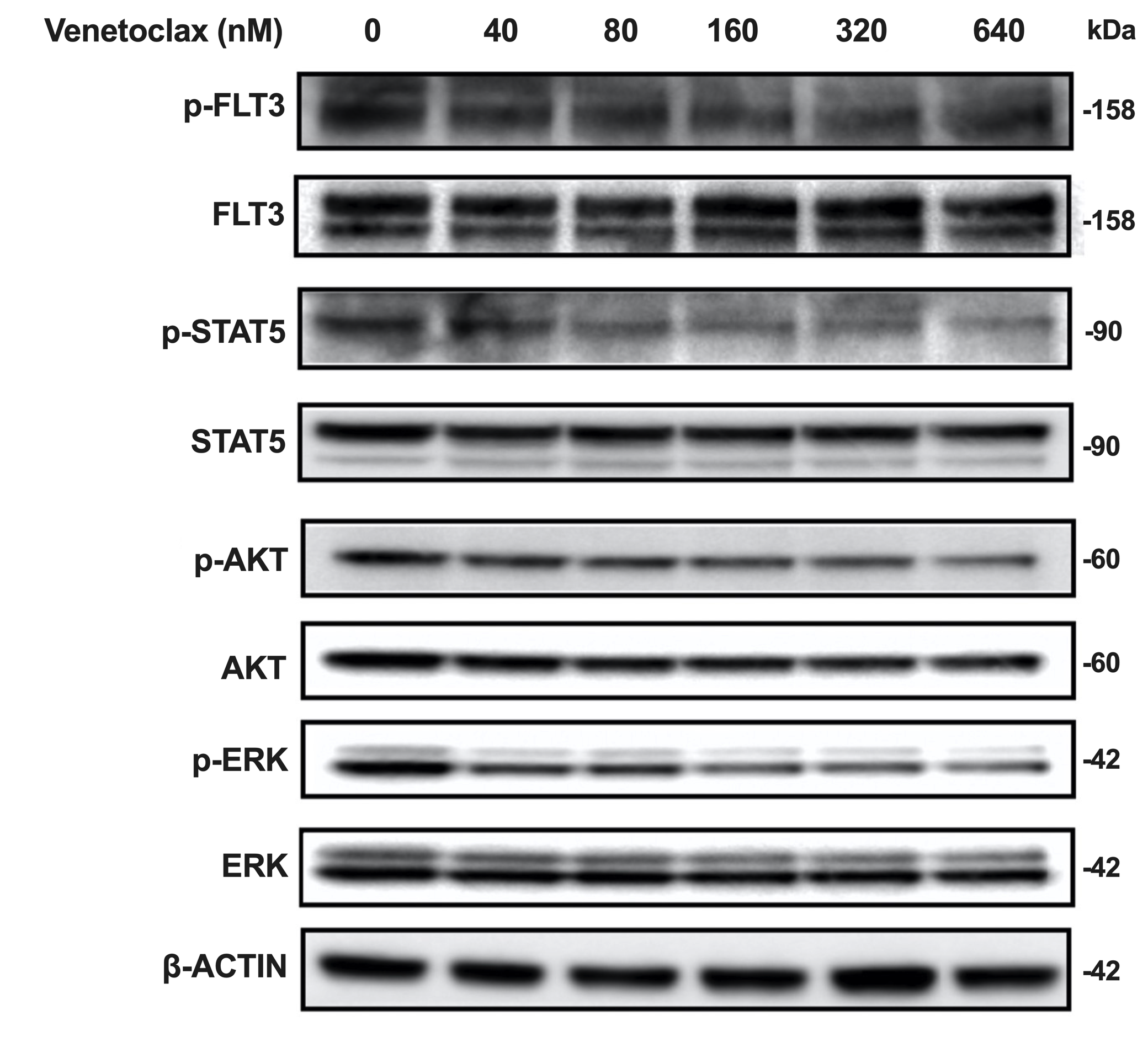


**Fig S10.** Western blot results for Molm14 cells treated with the indicated concentrations of Venetoclax alone.

| Cell line | CI Value |
| --- | --- |
| HL-60 | 7.832 |
| U937 | 9.35E+18 |
| THP-1 | 574.972 |

**Table S1.** Combination index (CI) value for THP-1, HL-60 and U937 cells treated with the combination. Concentrations for Gilteritinib and Venetoclax are the same as those used for the Molm14 cell line.
